# The modular chromosomal genomic plasticity mediating high level antibiotic resistance in eight clinical carbapenem-resistant * Acinetobacter baumannii* strains

**DOI:** 10.7717/peerj.21106

**Published:** 2026-04-28

**Authors:** Shuai Wang, Xinrou Zheng, Xueyun Geng, Ling Li, Mingyu Wang

**Affiliations:** State Key Laboratory of Microbial Technology, Shandong University, Qingdao, China

**Keywords:** *Acinetobacter baumannii*, Carbapenem resistance, Genomic plasticity, Multidrug resistance, Whole genome sequencing, Plasmid

## Abstract

**Objective:**

*Acinetobacter baumannii*, a prevalent multidrug resistant (MDR) pathogen, poses a significant threat to critically ill patients. This work aims to analyze the genomes of eight clinical carbapenem-resistant * A. baumannii* (CRAB) strains, and to study the mechanisms and genomic context of antimicrobial resistance for this critical pathogen.

**Methods:**

Nanopore whole-genomic sequencing was performed and compared with carbapenem-susceptible * A. baumannii* strain to identify genomic context patterns of antibiotic resistance.

**Results:**

Although some of these strains contain transferable plasmids, 121 of 122 antibiotic resistance genes (ARGs) identified are located on their chromosomes. Moreover, chromosomal ARGs clustered within recombinase-rich regions forming clear modules that are different from known resistance genomic islands. These modules are tandemly linked forming different combinations, and integrated at specific hot-spots *via* diverse mechanisms (direct repeats, fragment replacement, inverted repeats), suggesting that modular chromosomal plasticity leads to multidrug resistance in *A. baumannii*. Plasmid presence of these modules in other bacterial strains suggests they could have originated from plasmids.

**Conclusions:**

We find that modular chromosomal plasticity is the primary driver of carbapenem-resistance in our collection of CRAB isolates, which is a unique evolutionary strategy different from other ESKAPE pathogens. This study provides critical insights into CRAB genomic adaptability, and informs future strategies to combat their spread.

## Introduction

*Acinetobacter baumannii* is a commonly observed nosocomial bacterial pathogen that is well-known not just for its virulence, but more importantly for its high level of antimicrobial resistance (AMR) (Sharma & Lakhanpal, 2025). It is a member of ESKAPE (*Enterococcus faecium*, *Staphylococcus aureus*, *Klebsiella pneumoniae*, *Acinetobacter baumannii*, *Pseudomonas aeruginosa* and *Enterobacter* spp.), the six most concerned pathogens (Chong et al., 2025). Carbapenem-resistant *A. baumannii* (CRAB) is also among the three members of the critical group in World Health Organization’s Bacterial Priority Pathogens List of 2024 (Sati et al., 2025).

Clinically, *A. baumannii* often infects mucosa and skin (Nocera, Attili & De Martino, 2021), causing lower respiratory tract, wound, bloodstream, and urinary tract infections (Sharma & Lakhanpal, 2025). It is most notorious for its nosocomial dissemination among most critically-ill and immunity-weakened patients, particularly in intensive care units (ICUs) (Sharma & Lakhanpal, 2025). Surveillance studies showed a high CRAB prevalence of 71.4% in ICUs investigated in China (Liu et al., 2022; Jiang et al., 2022). A signature for *A. baumannii* in clinics is its high MDR levels. One study showed that the global prevalence of MDR among *A. baumannii* isolates causing hospital-acquired pneumonia (HAP) and ventilator-associated pneumonia (VAP) was 79.9% (95% Cl [73.9–85.4%]), and the mortality was estimated to be 42.6% (95% Cl [37.2–48.1%]) (Mohd Sazlly Lim et al., 2019).

The antibiotic resistance determinants for *A. baumannii* have been quite well studied, revealing a variety of acquired antibiotic resistance genes (ARGs) and mutations that are conserved among other bacteria (Lee et al., 2017). It has been well understood that uptake of antimicrobial resistance determinants by acquiring mobile genetic elements such as chromosomal resistance genomic islands and plasmids are common phenomenons, and these mechanisms often cause high level dissemination of AMR (Partridge et al., 2018). For many antibiotic resistant pathogens such as *K. pneumoniae* and *Escherichia coli*, acquiring antibiotic-resistant plasmids is the major mechanism for AMR, and most of the ARGs were plasmid-borne (Li et al., 2018; Wang et al., 2024; Xu et al., 2024). These transferrable ARGs were given more attention as they have a higher probability for fast dissemination than non-transferrable mechanisms such as resistance-conferring chromosomal mutations (Liu et al., 2016; Su et al., 2024). In *A. baumannii*, plasmids carrying ARGs have been identified, but it has been considered that chromosomal carriage of ARGs is a more common phenomenon (Tobin, Lam & Hamidian, 2025; Castillo-Ramírez et al., 2025).

The introduction of 3rd generation long-read sequencing drastically reduces difficulties in identifying and assembling plasmids (Wang et al., 2024; Xu et al., 2024), which now enables us to more easily understand the genomic location of ARGs. Using this technology, this work analyzed the high quality whole genomes of eight clinical *A. baumannii* strains. By doing this, this work attempts to reveal the patterns of *A. baumannii* to carry antimicrobial resistance, and to better understand the genomic context of ARGs of *A. baumannii*.

## Material and Methods

### Strains

*A. baumannii* strains were part of the bacterial collection of the Maternal and Child Health Hospital of Hubei Province, China (Table S1).

### Antibiotic susceptibility assays

Antibiotic susceptibility assays for ceftazidime (CAZ), cefepime (FEP), imipenem (IPM), and meropenem (MEM) were performed with VITEK 2 Compact system (bioMérieux, Inc., Durham, NC, USA), with the results interpreted as susceptible or resistant according to the Clinical and Laboratory Standards Institute (CLSI) M100 (32nd edition).

### Whole genome sequencing

Bacterial strains were plated, screened, and grown on LB media. Total DNA was extracted with the TIANamp Bacteria DNA Kit (DP302) from Tiangen biochemical technology (Beijing) Co., Ltd. (Beijing, China). Library was prepared with Rapid Barcoding Kit 96V14 (SQK-RBK114.96) for Nanopore sequencing. Third generation sequencing of genomes were performed with a Nanopore P2solo sequencer (Oxford Nanopore Technologies, Oxford, UK). The R10.4.1 flow cells were used for sequencing.

### Bioinformatics

Basecalling and demuxing to generate sequences for each strain was done with Dorado v0.7.4. Raw sequencing reads (FASTQ format) were quality-filtered and trimmed using Chopper v0.8.0 with the following parameters: minimum average quality score of 10 (-q 10), minimum read length of 500 bp (-l 500), and 50 bp removed from both ends of each read (–headcrop 50 –tailcrop 50). Clean sequences were subsequently assembled with Flye v2.8.1-b1676 with the –nano-hq –read-error 0.03 -t 64 parameter to generate whole genome sequences (Kolmogorov et al., 2019). Medaka v1.12.0 was used to correct the whole genomes. The specific model used was r1041 e82 400bps sup v4.3.0, which is a high-accuracy model. The assembled genomes were subsequently checked with BUSCO v5.2.2 for sequence completeness (Manni et al., 2021), Checkm2 v1.0.1 for sequence contamination (Parks et al., 2015), and Quast v5.0.2 for overall sequence quality (Mikheenko et al., 2018). The taxonomic classification was performed with GTDB-tk v2.1.1 (Chaumeil et al., 2022). The genomes were annotated with the Prokaryotic Genome Annotation Pipeline (Li et al., 2021). Identification of ARGs was performed with AMRfinder v3.11.26 (Feldgarden et al., 2019). The assembly graph from Flye was examined to confirm plasmid circularity, and plasmid copy numbers were estimated based on relative read coverage. All plasmid sequences were searched against the NT database of GenBank (accession date Feb. 24th, 2025) using BLASTn v2.14.0+ (Camacho et al., 2009). The plasmid search was conducted with an e-value of 10. Snippy v4.6.0 and Gubbins v2.4.1 were used for SNP analysis (Croucher et al., 2015). FastTree v2.1 was used for SNP-based phylogenetic analysis (Price, Dehal & Arkin, 2010). SnapGene v7.2.1 was used for sequence analysis and visualization. iTol v7.1.1 was used for phylogenetic tree visualization (Letunic & Bork, 2024). The structural modules were analyzed for mobile genetic elements using MobileElementFinder v1.1.2 with default parameters. Analyzed the sequencing results of the clinical isolates by comparing them with *Acinetobacter baumannii* ATCC19606.

### Multi-Locus Sequence Typing (MLST)

The MLST of the eight *A. baumannii* strains was analyzed using https://pubmlst.org/. The sequence types (STs) of the genomes were assigned according to the Pasteur MLST schemes. Core genome MLST (cgMLST) typing was performed *via* the cgMLST v1 on the PubMLST website.

## Results and Discussion

### Strains and whole genomic sequencing

Eight *A. baumannii* strains from the bacterial collection of the Maternal and Child Health Hospital of Hubei Province were studied in this work. These strains have nearly identical antibiotic resistance phenotypes (Table 1), showing resistance to ceftazidime (CAZ), cefepime (FEP), imipenem (IPM), meropenem (MEM). These strains are therefore classified as CRAB strains. The highly similar antibiotic resistance profiles prompted us to wonder whether they have similar antibiotic resistance mechanisms. Nanopore whole genome sequencing was subsequently performed to identify antibiotic resistance determinants for these strains, and to find out the genomic context patterns of antimicrobial resistance.

High quality whole genome sequences were assembled for all strains from Nanopore sequenced reads (Table 2). All chromosomes and all plasmids larger than 10 kb in size were circular, suggesting good assembly quality. Plasmids were found for all but one strains. An average of 2.4 ± 1.1 unique plasmids was found for each sequenced *A. baumannii* strain. A plasmid was considered to have a new structure if it exhibited less than 80% sequence identity and coverage compared to known plasmids. Based on this criterion, none of the plasmids found with whole genomic sequencing have new architectures.

The lack of new plasmids for *A. baumannii* strains differs from what have been observed for other common MDR pathogens like *K. pneumoniae* and *E. coli* (Wang et al., 2024; Xu et al., 2024). It was found that with Nanopore long-read sequencing and high quality assemblies, approximately half of the plasmids found have novel structures, which suggested the high plasticity for plasmids in these species. *A. baumannii*, however, appears to have quite stable plasmid structures, and hybridization and exchange of DNA between plasmids that lead to new plasmids seems to be rarer than other bacteria (Table 3). This could be the reason for the lack of plasmid-mediated antimicrobial resistance for *A. baumannii*, as recombination of plasmids appears to be slower.

**Table 1:** Antibiotic susceptibility of investigated *A. baumannii* strains.

**Strain**	**MIC (mg/L)**			
	CAZ	FEP	IPM	MEM
HB2490	≧64 (R)	≧32 (R)	≧16 (R)	≧16 (R)
HB2492	≧64 (R)	≧32 (R)	≧16 (R)	≧16 (R)
HB2496	≧64 (R)	≧32 (R)	≧16 (R)	≧16 (R)
HB2541	16 (R)	≧32 (R)	≧16 (R)	≧16 (R)
HB2548	≧64 (R)	≧32 (R)	≧16 (R)	≧16 (R)
HB2577	≧64 (R)	≧32 (R)	≧16 (R)	≧16 (R)
HB2581	≧64 (R)	≧32 (R)	≧16 (R)	≧16 (R)
HB2589	≧64 (R)	16 (R)	≧16 (R)	≧16 (R)

**Notes.**

CAZ ceftazidime FEP cefepime IPM imipenem MEM meropenem R resistant

**Table 2:** Whole genome features of *A. baumannii* strains.

Strain	Contig	Size (kb)	Molecule type	Cirularity	Copy number	Completeness	Contamination
HB2490	Contig 1	4,057.0	Chromosome	Yes	1	100	0.38
	Contig 2	19.8	Plasmid	Yes	4		
	Contig 3	73.2	Plasmid	Yes	1		
	Contig 4	111.0	Plasmid	Yes	1		
HB2492	Contig 1	4,010.1	Chromosome	Yes	1	100	0.21
HB2496	Contig 1	3.4	Plasmid	Yes	10	100	0.21
	Contig 3	3,963.3	Chromosome	Yes	1		
	Contig 4	6.03	Plasmid	No	10		
	Contig 5	71.3	Plasmid	Yes	1		
HB2541	Contig 1	8.7	Plasmid	Yes	16	100	0.25
	Contig 2	4,053.4	Chromosome	Yes	1		
	Contig 3	73.2	Plasmid	Yes	1		
HB2548	Contig 1	3,965.0	Chromosome	Yes	1	100	0.14
	Contig 2	78.0	Plasmid	Yes	1		
	Contig 3	17.5	Plasmid	Yes	3		
HB2577	Contig 1	6.3	Plasmid	No	14	100	0.37
	Contig 2	4,038.9	Chromosome	Yes	1		
	Contig 3	4.9	Plasmid	No	14		
	Contig 4	110.9	Plasmid	Yes	1		
HB2581	Contig 1	5.4	Plasmid	No	15	100	0.37
	Contig 2	4,038.9	Chromosome	Yes	1		
	Contig 3	110.8	Plasmid	Yes	1		
	Contig 4	5.8	Plasmid	No	15		
HB2589	Contig 1	4,040.1	Chromosome	Yes	1	100	0.36
	Contig 2	111.4	Plasmid	Yes	1		
	Contig 3	6.2	Plasmid	No	24		
	Contig 4	5.0	Plasmid	No	24		

**Table 3:** BLAST results of plasmids identified in the eight *A. baumannii* strains.

**Strain**	**Contig**	**Subject title**	**Identity (%)**	**Query coverage per subject (%)**
HB2490	Contig 2	CP121569.1*Acinetobacter baumannii* strain RAB73 plasmid pII_RAB73	100	100
	Contig 3	CP099785.1*Acinetobacter baumannii* strain NCCP 15989 plasmid unnamed	99.968	88
	Contig 4	CP121616.1*Acinetobacter baumannii* strain JAB108 plasmid pV_JAB108	99.993	99
HB2496	Contig 1	CP050524.1*Acinetobacter baumannii* strain VB7036 plasmid pVB7036_1	100	100
	Contig 4	MK531537.1*Acinetobacter baumannii* strain MC1/MC23 plasmid pMC1.2/pMC23.2	100	100
	Contig 5	CP104909.1*Acinetobacter baumannii* strain YZM-0314 plasmid punamed1	99.998	100
HB2541	Contig 1	CP121613.1*Acinetobacter baumannii* strain JAB117 plasmid pII_JAB117	100	100
	Contig 3	CP025267.1*Acinetobacter baumannii* isolate SMC_Paed_Ab_BL01 plasmid pSMC_AB_BL01_1	99.994	100
HB2548	Contig 2	MT802098.1*Acinetobacter baumannii* strain 2018HLJAB2 isolate 5630 plasmid unnamed	99.99	100
	Contig 3	CP121628.1*Acinetobacter baumannii* strain JAB144 plasmid pII_JAB144	100	100
HB2577	Contig 1	CP122366.1*Acinetobacter baumannii* strain SCCH66:Ab99738 plasmid pAb99738-1	100	100
	Contig 3	CP040086.1*Acinetobacter baumannii* strain VB33071 plasmid unnamed2	100	100
	Contig 4	CP077827.1*Acinetobacter baumannii* strain DETAB-E108 plasmid pDETAB12	99.987	100
HB2581	Contig 1	CP040086.1*Acinetobacter baumannii* strain VB33071 plasmid unnamed2	100	100
	Contig 3	CP077827.1*Acinetobacter baumannii* strain DETAB-E108 plasmid pDETAB12	99.987	100
	Contig 4	CP122366.1*Acinetobacter baumannii* strain SCCH66:Ab99738 plasmid pAb99738-1	100	100
HB2589	Contig 2	CP050908.1*Acinetobacter baumannii* strain DT-Ab022 plasmid unnamed1	99.871	100
	Contig 3	CP122366.1*Acinetobacter baumannii* strain SCCH66:Ab99738 plasmid pAb99738-1	100	100
	Contig 4	CP040086.1*Acinetobacter baumannii* strain VB33071 plasmid unnamed2	99.98	100

**Figure 1:**
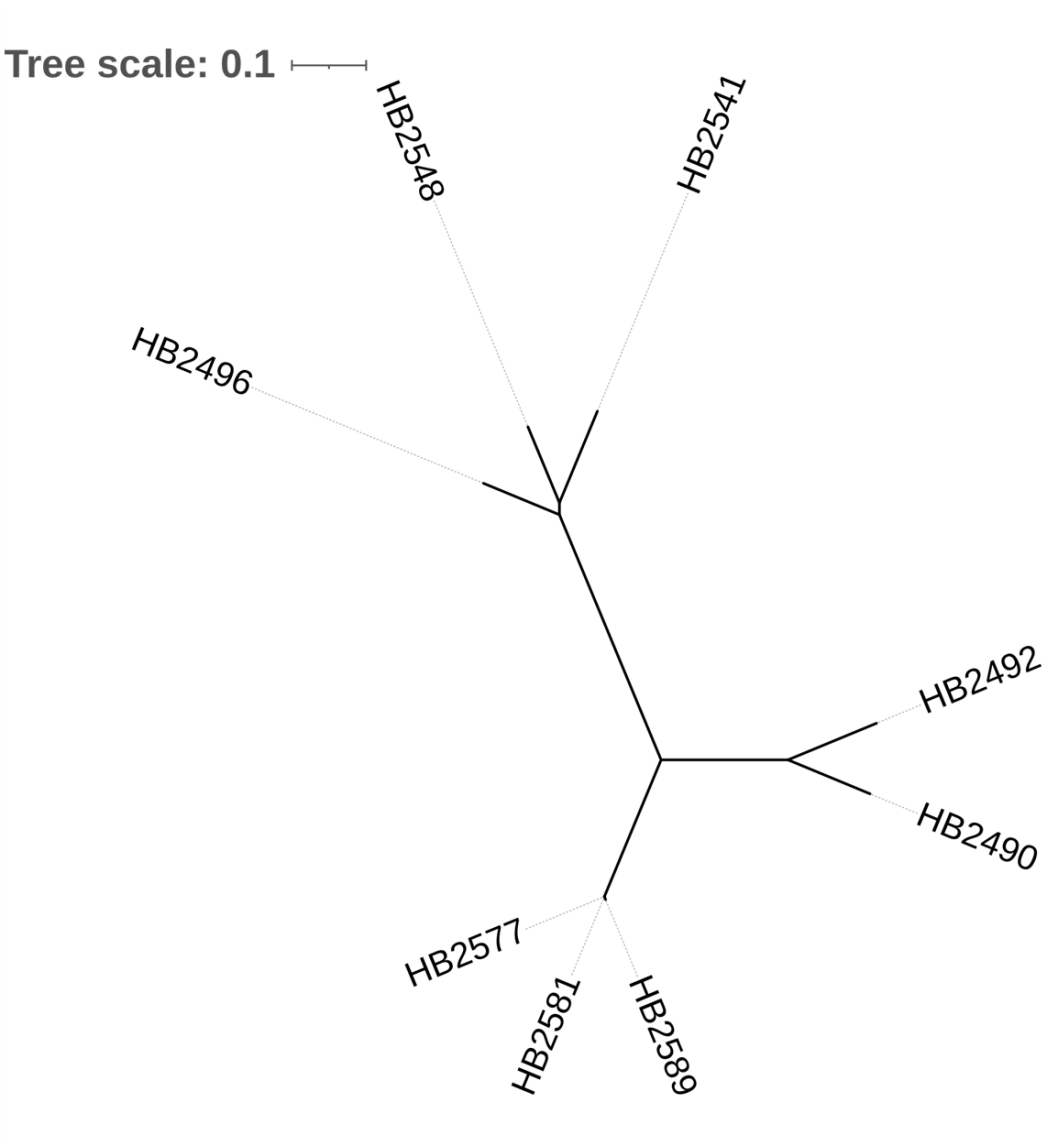
Phylogenetic analysis of *A. baumannii* strains. The phylogenetic tree was constructed using 242 SNP sites of the genomic sequences, identified with Snippy. The bar indicates evolutionary distance. Phylogenetic tree was constructed with FastTree. The reference genome was * A. baumannii* strain HB2490.

SNP-based evolutionary analysis showed that strains HB2581, HB2589, and HB2577 are closely related strains, with strains HB2577 and HB2581 being nearly identical (Fig. 1). All other strains showed marked phylogenetic differences, which formed distinct, well-separated clades on the phylogenetic tree, indicating substantial evolutionary divergence from the HB2581/HB2589/HB2577 group. This, together with their similar β-lactam and carbapenem resistance phenotypes, makes detailed comparison of antibiotic resistance mechanisms meaningful.

### ARGs in *A. baumannii* strains

The presence of ARGs were identified for sequenced *A. baumannii* strains. Of the 122 ARGs identified by AMRfinder, only one *bla*_OXA-23_ gene (for strain HB2548) was plasmid-borne (Table S2). Although some of the plasmids were clearly mobile because of the presence of transconjugation gene clusters, they do not carry ARGs. This is in stark contrast to *K. pneumoniae* and *E. coli*, for which most of the ARGs were present on plasmids (Li et al., 2018; Wang et al., 2020).

ARGs harbored by studied *A. baumannii* strains include those targeting β-lactam, carbapenem, aminoglycoside, tetracycline, macrolide, sulfonamide, chloramphenicol, and quaternary ammonium. The β-lactam resistance genes for each strain include one *bla*_TEM-1_ gene, and the distribution of *bla*_ADC_ variants is as follows: HB2490, HB2492, HB2577, HB2581, and HB2589 strains carry *bla*_ADC-30_ gene; HB2496 and HB2548 strains carry *bla*_ADC-73_ gene; whereas HB2541 strain lack any *bla*_ADC_ variant. The carbapenem resistance genes include 1-2 *bla*_OXA-23_ and one *bla*_OXA-66_. The strains carry 5-9 aminoglycoside resistance genes, including *aadA1*, *armA*, *ant* variants, *aph* variants, and *aac* variants. All strains carry a *tet(B)* tetracycline resistance gene. Half of the strains carry macrolide resistance genes *mph(E)* and *msr(E)*. For sulfonamide resistance genes, five strains carry 1-2 copies of *sul1*, and two strains carry two copies of *sul2* each. Every *sul1*-harboring strain carries a copy of *qacE*Δ*1*, indicative of an integron. Two different integron structures could be observed, with gene cassette arrays of *aac(6′)-Ib*-*catB8-aadA1* and *aac(3′)-Ia-orf1-orf2-aadA1*, respectively. Two strains carry chloramphenicol resistance *catB8*.

When compared with the CRABs in this work, the carbapenem-susceptible strain *A. baumannii* ATCC19606 was also found to carries a *bla*_ADC_ variant (*bla*_ADC-158_) and a *bla*_OXA_ variant (*bla*_OXA-98_). These two genes are conserved in the clinical CRABs studied in this work. The whole-genome MLST results for these *A. baumannii* strains indicated that all eight isolates belonged to the ST2 (Table 4). Notably, the cgST groupings aligned with the phylogenetic structure inferred from SNP analysis. Specifically, strains with nearly identical SNP profiles (HB2577, HB2581, HB2589, HB2492, HB2490) constituted one cgST group, while other, phylogenetically distinct strains formed separate cgST groups. This congruence confirms clonality at the sequence type level and delineates finer sub-lineage structures. The dominance of the ST2 clone among our isolates supports its status as a key epidemic lineage of CRAB, underscoring the need to monitor its transmission (Zhang et al., 2021; Mateo-Estrada et al., 2021).

**Table 4:** MLST analysis of the eight *A. baumannii* strains.

**ID**	**ST**	**cgST**	**CC**
HB2490	2	1,616	CC2
HB2492	2	1,616	CC2
HB2496	2	10,855	CC2
HB2541	2	4,200	CC2
HB2548	2	14,870	CC2
HB2577	2	1,638	CC2
HB2581	2	1,638	CC2
HB2589	2	1,638	CC2

**Notes.**

ST Sequence Type cgST Core Genome Sequence Type CC Clonal Complex

### Modular chromosomal plasticity leads to carbapenem-resistance in *A. baumannii* strains

**Figure 2:**
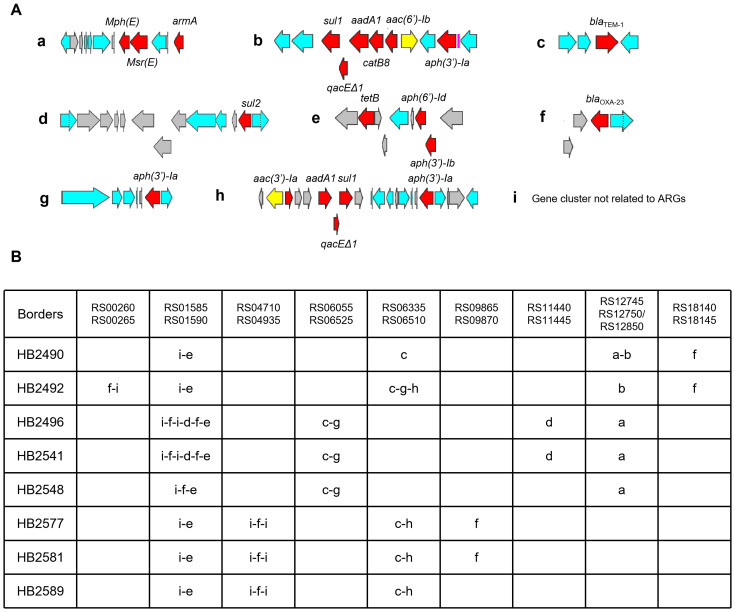
Antibiotic resistan ce modules and their location of integration. (A) structures of antibiotic resistance modules. Red color indicates antibiotic resistance genes, blue color indicates recombinase genes, yellow color indicates integron integrase genes, grey color indicates other genes. (B) location of integration and integrated antibiotic resistance modules. All changes were found when comparing with the genome of *A. baumannii* ATCC19606. Gene numbers are GenBank gene nomenclature for *A. baumannii* ATCC19606. For instance, RS00260 indicates gene FQU82_RS00260 in the GenBank database.

Further inspection of the chromosomal ARGs in the CRABs found that they are, with no exception, present in recombinase-rich gene modules (Fig. 2A). A total of eight such gene modules were identified in the strains. These gene modules were predominantly composed of transposases from the IS*26* and IS*Aba1* families. Additionally, they harbored transposases belonging to the IS*Vsa3*, IS*CR1*, IS*5*, IS*Ec29*, and IS*Aba24* families (Table S3). Despite the significant phylogenetic differences between the strains (Fig. 1), each gene module was present in at least two strains. They were also found in different locations in different strains (Fig. 2B). For instance, *bla*_TEM-1_-containing gene module c was found in two different locations for the eight strains. In three strains (HB2496, HB2541, HB2548), it was found to be co-integrated with gene module g. In three other strains (HB2577, HB2581, HB2589), it was found to be co-integrated with gene module h. In one strain HB2492, it was found to be co-integrated with both gene modules g and h. In the last strain HB2490, it was found to be integrated by itself.

The modular chromosomal ARG-containing gene clusters are integrated into *A. baumannii* chromosomes at specific hot-spots (Fig. 2B). A total of nine hot-spots were found. In all but one hot-spot, gene cluster integration took place in more than one strain. To preclude potential misassembly artifacts from gene duplication, we confirmed the presence of identical modules within individual genomes by PCR (Table S4 and Fig. S1). This further confirms the modularity of the ARG-containing gene clusters, as these clusters are not only modular by themselves, they are also integrated in different but specific spots. This modular chromosomal plasticity differed from several representative resistance genomic islands in *A. baumannii*, such as AbGRI1, AbGRI2, AbGRI3 and AGI1 (Blackwell, Nigro & Hall, 2015; Blackwell et al., 2017; Hamidian & Hall, 2017; Siebor & Neuwirth, 2020), as none of antimicrobial resistance modules bear structural similarities with these known resistance genomic islands.

The modular chromosomal plasticity for carbapenem-resistance in *A. baumannii* suggests a distinct resistance paradigm. In many bacterial pathogens such as *K. pneumoniae* and *E. coli* strains, acquisition of antibiotic resistant plasmids is the primary method for antibiotic resistance. This approach is highly efficient, as horizontal gene transfer normally happens at an appreciable rate, sometimes as high as 10^−6^. In contrast, chromosomal integration normally happens at a lower rate. Therefore, the strategy *A. baumannii* uses for antibiotic resistance is unusual, particularly considering that *A. baumannii* does uptake plasmids. Seven out of eight *A. baumannii* strains studied in this work contain plasmids, some of which contain transconjugation genes implying transferability.

### Characterization of hot-spots for chromosomal plasticity

**Figure 3:**
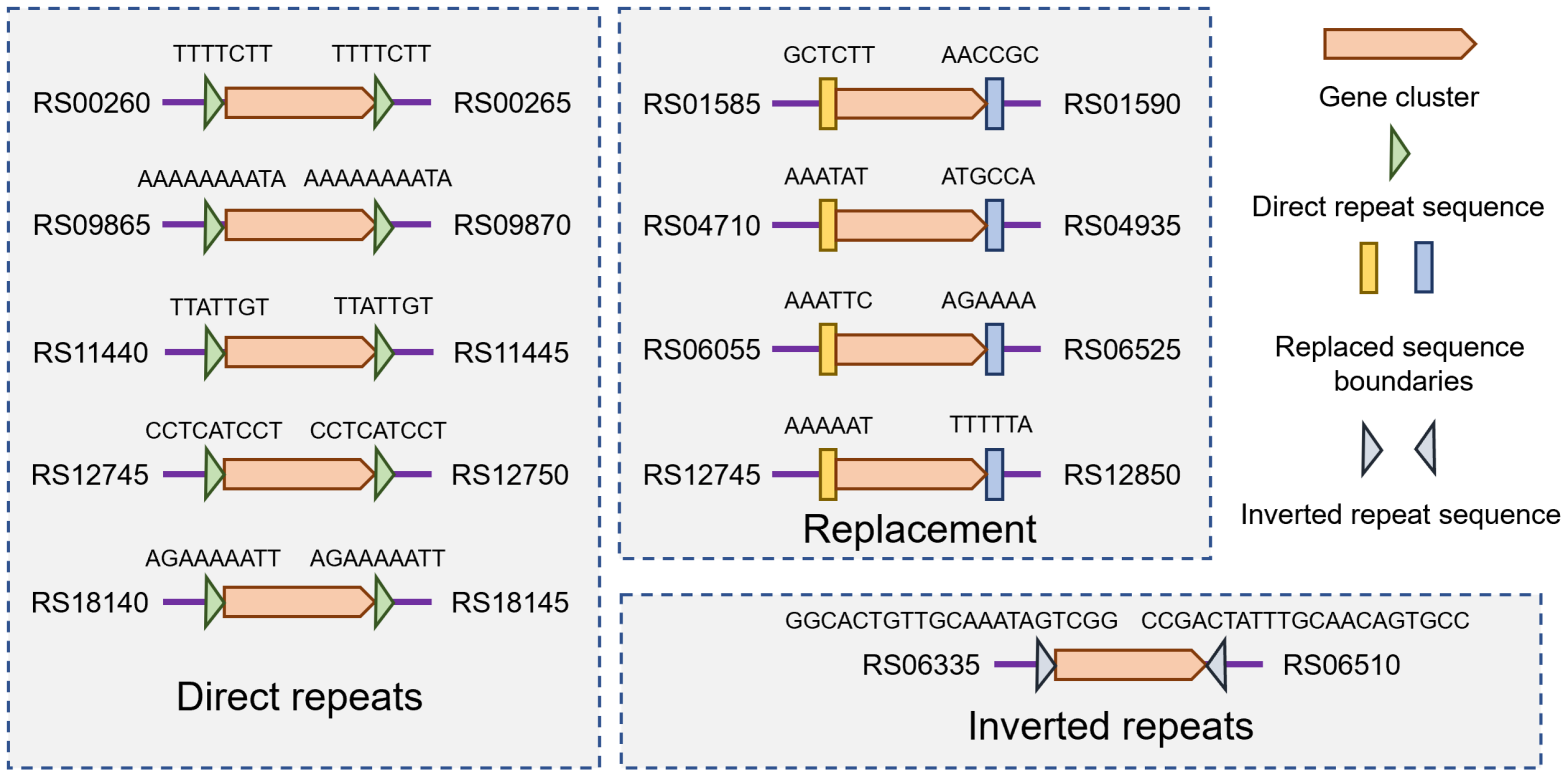
Organization of antibiotic gene clusters and bordering sequence features. Gene numbers are GenBank gene nomenclature for *A. baumannii* ATCC19606.

The hot-spots for antibiotic resistance gene cluster integration were analyzed for sequence properties. Three different types of integration were identified (Fig. 3). For five locations, modular gene clusters were bordered with two direct repeats that were present in the reference genome, suggesting integration events targeting the direct repeat sequences. For four locations, gene clusters replaced specific fragments of the *A. baumannii* chromosome, but no apparent sequence features could be observed. In one location, a fragment of *A. baumannii* chromosome was replaced with a gene cluster bordered with inverted repeats, suggested specific sequence-guided integration. It needs to be noted that at one location (Fig. 3, RS12745), two different mechanisms are present simultaneously.

The presence of three different types chromosomal gene cluster integration features indicate the diversity of chromosomal plasticity mechanisms, of which some are clearly recombinase-guided, whereas for the others specific mechanisms cannot be instantly recognized. Either way, for every type of integration, clear modularity is present, as different combinations of gene cluster modules were integrated in the same hot-spot with identical gene boundaries and integration locations. This suggests the modular chromosomal plasticity is caused by multiple mechanisms in *A. baumannii*.

The integrated gene modules are recombinase gene-rich (Fig. 2), which fits well with what is normally observed for plasmids (Badel, Da Cunha & Oberto, 2021). Whether these gene modules can be found on plasmids were studied by BLASTn search against the GenBank nt database, which led to the finding of nearly identical gene modules on plasmids for six of the eight gene clusters. Two of the plasmids were found in other species including *K. pneumoniae* and *Serratia marcescens* (Table 5). This finding, in combination with the finding that specific recombination mechanisms are involved in integration, led to the hypothesis that specific recombination mechanisms are involved in the modular chromosomal plasticity that results in CRAB: antibiotic resistant plasmids are acquired by *A. baumannii*, whose antibiotic gene modules are integrated into *A. baumannii* chromosome at specific hot-spots, with specific integration/recombination mechanisms. These mechanisms could be regulated by antibiotic stress and other environmental factors, and could lead to fast evolution of CRABs that are clinically dangerous. We believe further interrogation and elucidation of these carbapenem-resistant evolution paradigms could lead to methods to slow down or even prevent the emergence of CRAB.

**Table 5:** Identification of antibiotic resistance gene modules on plasmids.

**Gene module**	**GenBank Accession number**	**Description**
a	MN310378.1	*Klebsiella pneumoniae* strain A2293 plasmid pA2293-Ct2
b	CP077849.1	*Acinetobacter baumannii* strain DETAB-P16 plasmid pDETAB15
c	CP064203.1	*Acinetobacter baumannii* strain X4-300 plasmid unnamed1
d	AP025537.1	*Acinetobacter baumannii* OCU-Ac20 plasmid pOCUAc20-2 DNA
f	CP134588.1	*Acinetobacter baumannii* strain XH1026 plasmid pXH1026_2
h	KU315015.1	*Serratia marcescens* strain NCTC 50331 plasmid R1215

## Conclusions

Eight clinical carbapenem-resistant *A. baumannii* strains were analyzed for their genomic features. It is intriguing to find that all but one ARG is chromosomal. Further inspection of chromosomal regions with ARGs found that modular chromosomal plasticity is the primary reason for multidrug resistance: most ARGs are present in the form of antibiotic resistance modules, these modules are grouped and inserted into specific chromosomal hot-spots. We propose that these hotspots are mediated by enzymes specifically targeting the repeated sequences, despite the lack of a conserved target site. However, the exact mechanism has yet to be determined. These findings suggest that modular chromosomal plasticity contributes to carbapenem resistance in these CRAB strains, deepening our understanding of this critical nosocomial multidrug resistant pathogen. A notable observation from our data is the inverse correlation between ceftazidime susceptibility and the absence of a chromosomal ADC β-lactamase, as exemplified by isolate HB2541. This strain, which demonstrated a lower MIC to ceftazidime, was the only one among the set lacking an ADC enzyme (Table S2). This finding strongly suggests that the presence of the intrinsic ADC β-lactamase constitutes a fundamental baseline‘ level of resistance to this cephalosporin in our strain population. A limitation of this study is the relatively limited diversity of the bacterial sample set. While our analysis provides valuable insights into modular chromosomal plasticity, this constraint necessitates caution when extrapolating our specific findings to the broader species population. Future studies incorporating a more extensive and phylogenetically diverse collection of strains will be essential to confirm the generalizability of these observations.

##  Supplemental Information

10.7717/peerj.21106/supp-1 Supplemental Information 1 Agarose gel electrophoresis of PCR validation for the presence of the antibiotic resistance modules

10.7717/peerj.21106/supp-2 Supplemental Information 2 Details of the eight clinical MDR * A. baumannii* strains collection

10.7717/peerj.21106/supp-3 Supplemental Information 3 Antibiotic resistance genes in each * A. baumannii* strain

10.7717/peerj.21106/supp-4 Supplemental Information 4 Functional annotation of genes in the antibiotic resistance modules (Figure 2)

10.7717/peerj.21106/supp-5 Supplemental Information 5 Primers for PCR validation of the antibiotic resistance modules discovered through sequencing

## Additional Information and Declarations

### Data Availability

The following information was supplied regarding data availability: The genomes are available at GenBank: PRJNA1259187 and PRJNA1259196.
