# Supplementary figures and images for: The modular chromosomal genomic plasticity mediating high level antibiotic resistance in eight clinical carbapenem-resistant Acinetobacter baumannii strains
Source: PeerJ. 2026 Apr 28;14:e21106. doi: 10.7717/peerj.21106 (PMC13134544; doi:10.7717/peerj.21106)

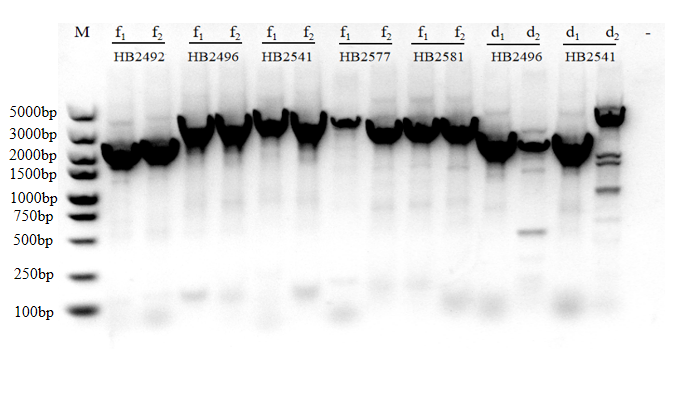

Supplement: Supplemental Information 1 [file peerj-14-21106-s001.png]
