# Supplementary material for: The modular chromosomal genomic plasticity mediating high level antibiotic resistance in eight clinical carbapenem-resistant Acinetobacter baumannii strains
Source: PeerJ. 2026 Apr 28;14:e21106. doi: 10.7717/peerj.21106 (PMC13134544; doi:10.7717/peerj.21106)
Supplement: Supplemental Information 2 [file peerj-14-21106-s002.docx]

**Table S1. Details of the eight clinical MDR *A. baumannii* strains collection.**

| **Strain** | **Sample type** | **Collection Date** | **Subculture Date** | **Cryopreservation Date** |
| --- | --- | --- | --- | --- |
| HB2490 | bronchoalveolar lavage fluid | 2023.01.31 | 2024.10.28 | 2024.10.29 |
| HB2492 | distal end of the endotracheal tube | 2023.02.07 | 2024.10.28 | 2024.10.29 |
| HB2496 | wound exudate | 2023.02.18 | 2024.10.28 | 2024.10.29 |
| HB2541 | sputum | 2023.07.06 | 2024.10.28 | 2024.10.29 |
| HB2548 | blood | 2023.07.21 | 2024.10.28 | 2024.10.29 |
| HB2577 | distal tip of the PICC catheter | 2023.10.29 | 2024.10.28 | 2024.10.29 |
| HB2581 | distal end of the endotracheal tube | 2023.12.17 | 2024.10.28 | 2024.10.29 |
| HB2589 | distal end of the endotracheal tube | 2024.01.25 | 2024.10.28 | 2024.10.29 |
