# Supplementary material for: The modular chromosomal genomic plasticity mediating high level antibiotic resistance in eight clinical carbapenem-resistant Acinetobacter baumannii strains
Source: PeerJ. 2026 Apr 28;14:e21106. doi: 10.7717/peerj.21106 (PMC13134544; doi:10.7717/peerj.21106)
Supplement: Supplemental Information 3 [file peerj-14-21106-s003.docx]

**Table S2. Antibiotic resistance genes in each *A. baumannii* strain.**

| **Strain** | **β-lactam** | **Carbapenem** | **Aminoglycoside** | **Tetracycline** | **Macrolide** | **Sulfonamide** | **Chloramphenicol** | **Quaternary ammonium** |
| --- | --- | --- | --- | --- | --- | --- | --- | --- |
| HB2490 | *bla*_ADC-30_  *bla*_TEM-1_ | *bla*_OXA-23_  *bla*_OXA-66_ | *ant(3’)-IIa*  *aph(6’)-Id*  *aph(3’)-Ib*  *armA*  *aadA1*  *aac(6’)-Ib*  *aph(3’)-Ia* | *tet(B)* | *mph(E)*  *msr(E)* | *sul1* | *catB8* | *qacEΔ1* |
| HB2492 | *bla*_TEM-1_  *bla*_ADC-30_ | *bla*_OXA-66_  *bla*_OXA-23_  *bla*_OXA-23_ | *aadA1*  *aac(3’)-Ia*  *aph(3’)-Ia*  *aph(3’)-Ib*  *aph(6’)-Id*  *ant(3’)-IIa*  *aph(3’)-Ia*  *aac(6’)-Ib*  *aadA1* | *tet(B)* |  | *sul1*  *sul1* | *catB8* | *qacEΔ1*  *qacEΔ1* |
| HB2496 | *bla*_ADC-73_  *bla*_TEM-1_ | *bla*_OXA-66_  *bla*_OXA-23_  *bla*_OXA-23_ | *armA*  *aph(3’)-Ia*  *aph(3’)-Ib*  *aph(6’)-Id*  *ant(3’)-IIa* | *tet(B)* | *mph(E)*  *msr(E)* | *sul2*  *sul2* |  |  |
| HB2541 | *bla*_TEM-1_ | *bla*_OXA-23_  *bla*_OXA-23_  *bla*_OXA-66_ | *armA*  *ant(3’)-IIa*  *aph(6’)-Id*  *aph(3’)-Ib*  *aph(3’)-Ia* | *tet(B)* | *mph(E)*  *msr(E)* | *sul2*  *sul2* |  |  |
| HB2548 | *bla*_ADC-73_  *bla*_TEM-1_ | *bla*_OXA-23_  *bla*_OXA-66_  *bla*_OXA-23_ | *aph(3’)-Ib*  *aph(6’)-Id*  *ant(3’)-IIa*  *armA*  *aph(3’)-Ia* | *tet(B)* | *mph(E)*  *msr(E)* |  |  |  |
| HB2577 | *bla*_ADC-30_  *bla*_TEM-1_ | *bla*_OXA-23_  *bla*_OXA-23_  *bla*_OXA-66_ | *ant(3’)-IIa*  *aph(6’)-Id*  *aph(3’)-Ib*  *aac(3’)-Ia*  *aadA1*  *aph(3’)-Ia* | *tet(B)* |  | *sul1* |  | *qacEΔ1* |
| HB2581 | *bla*_TEM-1_  *bla*_ADC-30_ | *bla*_OXA-23_  *bla*_OXA-23_  *bla*_OXA-66_ | *aph(6’)-Id*  *aph(3’)-Ib*  *aac(3’)-Ia*  *aadA1*  *aph(3’)-Ia*  *ant(3’)-IIa* | *tet(B)* |  | *sul1* |  | *qacEΔ1* |
| HB2589 | *bla*_ADC-30_  *bla*_TEM-1_ | *bla*_OXA-23_  *bla*_OXA-23_  *bla*_OXA-66_ | *ant(3’)-IIa*  *aph(6’)-Id*  *aph(3’)-Ib*  *aac(3’)-Ia*  *aadA1*  *aph(3’)-Ia* | *tet(B)* |  | *sul1* |  | *qacEΔ1* |
