# Supplementary material for: The modular chromosomal genomic plasticity mediating high level antibiotic resistance in eight clinical carbapenem-resistant Acinetobacter baumannii strains
Source: PeerJ. 2026 Apr 28;14:e21106. doi: 10.7717/peerj.21106 (PMC13134544; doi:10.7717/peerj.21106)
Supplement: Supplemental Information 4 [file peerj-14-21106-s004.docx]

**Table S3. Functional annotation of genes in the antibiotic resistance modules (Figure 2).**

|  | **Gene** | **Description** |
| --- | --- | --- |
| **a** | pgaptmp_003828 | IS*6*-like element IS*26* family transposase |
|  | pgaptmp_003831 | IS*66* family insertion sequence element accessory protein TnpB |
|  | pgaptmp_003832 | IS*66* family insertion sequence element accessory protein TnpB |
|  | pgaptmp_003833 | IS*66*-like element IS*Aba24* family transposase |
|  | *Mph(E)* | Mph(E) family macrolide 2'-phosphotransferase |
|  | *Msr(E)* | ABC-F type ribosomal protection protein Msr(E) |
|  | pgaptmp_003837 | IS*4*-like element IS*Ec29* family transposase |
|  | *armA* | 16S rRNA (guanine(1405)-N(7))-methyltransferase ArmA |
| **b** | pgaptmp_003840 | IS*5* family transposase |
|  | pgaptmp_003841 | IS*91*-like element IS*CR1* family transposase |
|  | *sul1* | sulfonamide-resistant dihydropteroate synthase Sul1 |
|  | *qacE∆1* | quaternary ammonium compound efflux SMR transporter QacE delta 1 |
|  | *aadA1* | ANT(3'')-Ia family aminoglycoside nucleotidyltransferase AadA1 |
|  | *catB8* | type B-3 chloramphenicol O-acetyltransferase CatB8 |
|  | *aac(6’)-lb* | aminoglycoside N-acetyltransferase AAC(6')-Ib' |
|  | *intI1* | class 1 integron integrase IntI1 |
|  | pgaptmp_003848 | IS*6*-like element IS*26* family transposase |
|  | *aph(3’)-la* | aminoglycoside O-phosphotransferase APH(3')-Ia |
|  | pgaptmp_003850 | IS*6* family transposase |
|  | pgaptmp_003851 | IS*6*-like element IS*26* family transposase |
| **c** | pgaptmp_002679 | IS*6*-like element IS*26* family transposase |
|  | *bla*_TEM-1_ | broad-spectrum class A beta-lactamase TEM-1 |
|  | pgaptmp_002680 | recombinase family protein |
|  | pgaptmp_002682 | IS*6*-like element IS*26* family transposase |
| **d** | pgaptmp_001119 | IS*4*-like element IS*Aba1* family transposase |
|  | pgaptmp_001111 | Mu transposase C-terminal domain-containing protein |
|  | pgaptmp_001110 | heteromeric transposase endonuclease subunit TnsA |
|  | *sul2* | sulfonamide-resistant dihydropteroate synthase Sul2 |
|  | pgaptmp_001107 | IS*4*-like element IS*Aba1* family transposase |
| **e** | *tet(B)* | tetracycline efflux MFS transporter Tet(B) |
|  | pgaptmp_001637 | IS*91*-like element IS*Vsa3* family transposase |
|  | *aph(6’)-ld* | aminoglycoside O-phosphotransferase APH(6)-Id |
|  | *aph(3’)-lb* | aminoglycoside O-phosphotransferase APH(3'')-Ib |
| **f** | *bla*_OXA-23_ | OXA-23 family carbapenem-hydrolyzing class D beta-lactamase OXA-23 |
|  | pgaptmp_001175 | IS*4*-like element IS*Aba1* family transposase |
| **g** | pgaptmp_002010 | IS*6*-like element IS*26* family transposase |
|  | pgaptmp_002009 | Tn3 family transposase |
|  | pgaptmp_002008 | recombinase family protein |
|  | pgaptmp_002007 | IS*6*-like element IS*26* family transposase |
|  | *aph(3’)-la* | aminoglycoside O-phosphotransferase APH(3')-Ia |
|  | pgaptmp_002004 | IS*6*-like element IS*26* family transposase |
| **h** | *intI1* | class 1 integron integrase IntI1 |
|  | *aac(3’)-la* | aminoglycoside N-acetyltransferase AAC(3)-Ia |
|  | *aadA1* | ANT(3'')-Ia family aminoglycoside nucleotidyltransferase AadA1 |
|  | *qacE∆1* | quaternary ammonium compound efflux SMR transporter QacE delta 1 |
|  | *sul1* | sulfonamide-resistant dihydropteroate synthase Sul1 |
|  | pgaptmp_002445 | IS*6* family transposase |
|  | pgaptmp_002446 | IS*6*-like element IS*26* family transposase |
|  | pgaptmp_002447 | recombinase family protein |
|  | pgaptmp_002449 | IS*6*-like element IS*26* family transposase |
|  | pgaptmp_002450 | IS*6* family transposase |
|  | *aph(3’)-la* | aminoglycoside O-phosphotransferase APH(3')-Ia |
|  | pgaptmp_002453 | IS*6*-like element IS*26* family transposase |
|  | pgaptmp_002456 | IS*6*-like element IS*26* family transposase |
