# Supplementary material for: The modular chromosomal genomic plasticity mediating high level antibiotic resistance in eight clinical carbapenem-resistant Acinetobacter baumannii strains
Source: PeerJ. 2026 Apr 28;14:e21106. doi: 10.7717/peerj.21106 (PMC13134544; doi:10.7717/peerj.21106)
Supplement: Supplemental Information 5 [file peerj-14-21106-s005.docx]

**Table S4. Primers for PCR validation of the antibiotic resistance modules discovered through sequencing.**

| **Primer** | **Sequence (5’→3’)** |
| --- | --- |
| HB2490-f_1_-F | CCGCCTATCAGTTCTCTA |
| HB2490-f_1_-R | ATGCTCCACTTGCCAGTC |
| HB2490-f_2_-F | CGTGGTCAACGGTCAGGA |
| HB2490-f_2_-R | GCCATTCCACTGCCAATA |
| HB2496-f_1_-F | GAGACACGCCAAAAAGCT |
| HB2496-f_1_-R | GAGAAGCAACCACATAGC |
| HB2496-f_2_-F | CAGGAGACAAGTTGTCAG |
| HB2496-f_2_-R | CTGACTCGGGGTTTCATT |
| HB2541-f_1_-F | GAACAATCTGACTCGGGG |
| HB2541-f_1_-R | CGATGCCATTGCAGCATT |
| HB2541-f_2_-F | GAGAAGCAACCACATAGC |
| HB2541-f_2_-R | GGGAGTAAACAGGAGACA |
| HB2577-f_1_-F | CTGGGAGCTTTTGTGAAA |
| HB2577-f_1_-R | GCAGGGGAAAGCTGAATT |
| HB2577-f_2_-F | CCCGCCATATACACGAAA |
| HB2577-f_2_-R | CTTCAATTGTCCGTGCCC |
| HB2581-f_1_-F | CCCGCCATATACACGAAA |
| HB2581-f_1_-R | CGGCACAGGAAAATCTTC |
| HB2581-f_2_-F | CGGTCCTTGCTATGACAT |
| HB2581-f_2_-R | GATTGTTGAGTTGGTACC |
| HB2541-d_1_-F | GTATGAGACTCATGCTCG |
| HB2541-d_1_-R | TTGCCACGAATACCGTCT |
| HB2541-d_2_-F | CCTGCTGAACCGCGAATA |
| HB2541-d_2_-R | GGGATAGTAGGAGCGTCA |
| HB2496-d_1_-F | AGGGGACATGCCTGCTGA |
| HB2496-d_1_-R | CCGTATGAGACTCATGCT |
| HB2496-d_2_-F | GTTAAGAGAAGCGCGACT |
| HB2496-d_2_-R | AAAGGGGACATGCCTGCT |
